# Association Between Nutrition, Diet Quality, Dietary Patterns, and Human Health and Diseases

**DOI:** 10.3390/nu17010003

**Published:** 2024-12-24

**Authors:** Jean L. Fry, Kayla R. Anderson

**Affiliations:** Department of Athletic Training and Clinical Nutrition, University of Kentucky, Lexington, KY 40536-0200, USA

Establishing relationships between diet and human health is an active and critical area of research, and this Special Issue, “Association Between Nutrition, Diet Quality, Dietary Patterns, and Human Health and Diseases”, is a collection of research highlighting classic and emerging themes in this area. This issue included 12 articles, selected from 40 initial submissions, covering topics, including how diet quality and dietary patterns influence chronic diseases, metabolic health, and human behavior across diverse populations. National nutrition guidelines, including the 2020–2025 Dietary Guidelines for Americans (DGFA) [1] and the U.S. National Institutes for Health (NIH) Strategic Plan for Nutrition Research [2], emphasize the importance of diet in human health. The DGFA highlights the role of nutrient-dense food choices and balance across food groups to prevent chronic diseases. Similarly, the NIH Strategic Plan defines strategic goals for research to understand how diet modulates disease burden to enable the development of innovative dietary interventions. Globally, organizations like the World Health Organization [3] and the Food and Agriculture Organization [4] promote healthy and environmentally sustainable diets to support global nutrition security and overall health. Collectively, these organizations reflect a growing acknowledgment of the critical link between diet and long-term health outcomes. Here, we summarize overarching themes and key findings in this Special Issue, as well as directions for future research.

One recurring theme in this Special Issue concerns the benefits of well-defined diets (Contributors 1–5). For example, research on the DASH diet, when combined with regular physical activity, demonstrated significant improvements in fat mass and cardiometabolic parameters in individuals with obesity (Contributor 4). Similarly, adherence to a traditional Mexican diet was associated with lower hepatic steatosis in U.S.-born people of Mexican descent. These findings highlighted the health benefits of traditional diets and the importance of culturally tailored diets (Contributor 3). Another study confirmed that participants consuming plant-based diets were at reduced cardiovascular disease risk compared to those consuming a diet high in meat products (Contributor 1). Collectively, these studies underscored the critical role of diet in chronic disease prevention and management.

Another key theme emerging from this Special Issue is the recognition of sex-specific differences in dietary responses (Contributors 6 and 7). The NIH Policy on Sex as a Biological Variable, released in 2016, mandates that scientists receiving NIH funding consider the biological sex of study participants. Moving forward, this policy will continue to advance research defining how biological sex influences responses to dietary patterns, nutrient absorption, metabolism, and disease susceptibility. In this Special Issue, a review highlighted metabolic variations between men and women in response to diet, emphasizing the need for clinical trials designed to test sex differences and facilitate precision dietary recommendations (Contributor 6). Another study reported sex-modified associations between BMI, food intake, and physical activity. The findings showed that both diet and physical activity excessively contribute to high BMI in young men, but this relationship was not observed in young women (Contributor 7). These articles highlighted the complexity of designing effective nutritional interventions that address biological and behavioral differences among the sexes.

Cultural and socioeconomic factors are also prominently featured in this edition (Contributors 3 and 8). This Special Issue included reports of culturally relevant diets, such as the traditional Mexican diet (Contributor 3). Studies also highlighted specific ethnic groups. For example, one study reported on the grocery shopping habits of African Americans with food insecurity (Contributor 8) to emphasize the role of socioeconomic factors in shaping diet quality and accessibility.

The NIH Strategic Plan for Nutrition Research challenges the science community to define the health-related effects of time-restricted eating, which includes interventions like intermittent fasting and alignment of eating with circadian rhythms. These innovative dietary interventions show promise in improving cardiometabolic markers in individuals with metabolic syndrome (Contributor 9). In this Special Issue, another report investigated the association between periodic fasting and acute cardiac events (Contributor 10). These studies contribute to a growing body of literature exploring the importance of meal timing for metabolic health. This line of inquiry is expected to determine the efficacy of time-restricted eating for improving chronic disease-associated health indicators while elucidating underlying mechanisms driving these outcomes.

Finally, this Special Issue highlighted the importance of nutrition across the lifespan (Contributors 7, 11, 12), another research priority the NIH highlighted. The NIH identified pregnancy, infancy, childhood, and older adulthood as critical life phases, and additional research is needed to clarify how eating behaviors should adapt over time to promote optimal health outcomes. In our Special Issue, a study focusing on childhood nutrition underscored the value of proper dietary practices during the first two years of life (Contributor 12), while another linked protein intake to Alzheimer’s disease outcomes in older adults (Contributor 11). These findings emphasize the importance of dietary considerations from infancy to advanced age.

The articles included in this Special Issue collectively highlighted the critical interplay between diet quality, metabolic health, and chronic disease prevention. They emphasize the necessity of recognizing sex-specific and cultural differences in dietary behaviors, addressing socioeconomic barriers to healthy eating, and developing evidence-based dietary guidelines that account for individual and environmental differences. Guidelines that consider individual differences, such as lifestyle factors and health conditions, alongside environmental influences, like food accessibility and cultural norms, are essential for advancing public health. Collectively, guidance relating to key topics in this issue will lay down a foundation for precision nutrition. More tailored dietary guidance will enable policymakers to create equitable food policies and clinicians to design tailored dietary recommendations. Together, these efforts can transform public health strategies and reduce the burden of diet-related chronic diseases.

Future research should focus on several key areas. Longitudinal studies are needed to establish causal relationships between dietary patterns and chronic diseases across diverse ethnic and clinical populations. By including more diverse populations over time, researchers can support the development of dietary guidelines and interventions with greater generalizability while also identifying sub-groups benefitting from tailored dietary guidance. Such targeted interventions are expected to optimize health outcomes, reduce the risk of chronic diseases, and support healthy aging. Additionally, researchers should prioritize the development of precision dietary interventions that consider socioeconomic and cultural contexts to improve dietary adherence and health outcomes. Considering the interplay of culture, socioeconomic factors, and diet will inform interventions that improve health outcomes across diverse populations. Moreover, mechanistic studies will be crucial for elucidating the biological pathways influenced by culturally specific diets, time-based approaches, and sex-based differences. Understanding the biological underpinnings of how diet affects chronic disease-related outcomes may uncover novel therapeutic targets.

In conclusion, this Special Issue underscored the profound impact of diet quality and patterns on human health and chronic disease prevention. The collective efforts of the contributing authors advanced our understanding of these relationships and confirmed previously reported relationships, laying the groundwork for future research and public health initiatives. I would like to thank all the authors, reviewers, and the editorial team for their contributions to the work needed to develop and publish this Special Issue.
